# Development of Pandemic Vaccines: ERVEBO Case Study

**DOI:** 10.3390/vaccines9030190

**Published:** 2021-02-25

**Authors:** Jayanthi Wolf, Risat Jannat, Sheri Dubey, Sean Troth, Matthew T. Onorato, Beth-Ann Coller, Mary E. Hanson, Jakub K. Simon

**Affiliations:** 1 Regulatory Affairs, Merck & Co. Inc., Kenilworth, NJ 07033, USA; jayanthiwolf@gmail.com; 2 Global Vaccines & Biologics Commercialization, Merck & Co. Inc., Kenilworth, NJ 07033, USA; risat_jannat@merck.com; 3 Pharmacokinetics, Pharmacodynamics & Drug Metabolism, Merck & Co. Inc., Kenilworth, NJ 07033, USA; sheri_dubey@merck.com; 4 Department of Safety Assessment and Laboratory Animal Resources, Merck & Co. Inc., Kenilworth, NJ 07033, USA; sean_troth@merck.com; 5 Global Clinical Trial Operations, Vaccines, Merck & Co. Inc., Kenilworth, NJ 07033, USA; matthew_onorato@merck.com; 6 Global Clinical Development, Vaccines, Merck & Co. Inc., Kenilworth, NJ 07033, USA; Beth-ann.coller@merck.com; 7 Global Scientific & Medical Publications, Merck & Co. Inc., Kenilworth, NJ 07033, USA; mary.hanson@merck.com

**Keywords:** *Ebolavirus* vaccine, rVSVΔG-ZEBOV-GP, vaccine development, regulatory strategy, vaccine manufacturing

## Abstract

Preventative vaccines are considered one of the most cost-effective and efficient means to contain outbreaks and prevent pandemics. However, the requirements to gain licensure and manufacture a vaccine for human use are complex, costly, and time-consuming. The 2013–2016 Ebola virus disease (EVD) outbreak was the largest EVD outbreak to date and the third Public Health Emergency of International Concern in history, so to prevent a pandemic, numerous partners from the public and private sectors combined efforts and resources to develop an investigational *Zaire ebolavirus* (EBOV) vaccine candidate (rVSVΔG-ZEBOV-GP) as quickly as possible. The rVSVΔG-ZEBOV-GP vaccine was approved as ERVEBO^TM^ by the European Medicines Authority (EMA) and the United States Food and Drug Administration (FDA) in December 2019 after five years of development. This review describes the development program of this EBOV vaccine, summarizes what is known about safety, immunogenicity, and efficacy, describes ongoing work in the program, and highlights learnings applicable to the development of pandemic vaccines.

## 1. Introduction

*Zaire ebolavirus* (EBOV) belongs to the *Filoviridae* family, a group of filamentous, enveloped RNA viruses that are maintained in nature in an enzootic cycle believed to involve bats, and epizootic cycles involving human and non-human primates (NHPs). The virus is transmitted through human-to-human contact after its introduction from the enzootic cycle [1]. EBOV disease (EVD) outbreaks cause significant human and societal impacts, including high mortality rates and long-term persistence in immune-privileged body sites in survivors [2]. Indirect health effects for those not infected include a reduction in uptake of reproductive, maternal, and child health services, human immunodeficiency virus (HIV)/acquired immunodeficiency syndrome care, and malaria services [3]. The largest EVD outbreak in the Democratic Republic of the Congo (DRC) in 2013–2016 was the third Public Health Emergency of International Concern ever declared in history and prompted intensified vaccine and antiviral development. 

**Figure 1:**
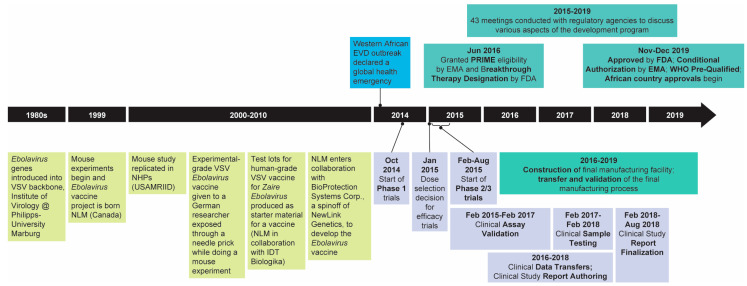
Timeline of *Zaire Ebolavirus* vaccine development beginning with *Ebolavirus* gene introduction into the VSV backbone in the 1980s through the European Medicines Agency (EMA) and the United States Food and Drug Administration (FDA) approval, WHO prequalification, and approval in African countries in 2019. EMA = European Medicines Agency; EVD = *Zaire ebolavirus* disease; FDA = United States Food and Drug Administration; NHPs = non-human primates; NLM = National Laboratory of Microbiology; USAMRIID = United States Army Medica Research Institute of Infectious Diseases; VSV = vesicular stomatitis virus; WHO = World Health Organization. Adapted from [7].

The typical development program for a vaccine takes more than 10 years and can cost more than USD 2 billion [4,5,6]. The rVSVΔG-ZEBOV-GP (ERVEBO^TM^) vaccine is a live, attenuated, recombinant vesicular stomatitis virus (rVSV)-based, chimeric-vector vaccine for which the VSV envelope protein was deleted and replaced (ΔG) by inserting only the envelope glycoprotein (GP) of EBOV. There is no live EBOV in the vaccine. Efforts to rapidly develop ERVEBO^TM^ resulted in the first regulatory approval just five years after the program was acquired (Figure 1) by Merck Sharp & Dohme Corp., a subsidiary of Merck & Co., Inc., Kenilworth, NJ, USA (MSD). 

## 2. Regulatory Considerations 

ERVEBO^TM^ was the first EBOV vaccine approved in the US, European Union, in several African countries, and to obtain prequalification by the World Health Organization (WHO) based on human efficacy data [8,9,10,11]. WHO prequalification process ensured that the vaccine met global standards of quality, safety, and efficacy and determines the acceptability of vaccines from different sources for supply to agencies such as UNICEF and other UN agencies. Three additional *Ebolavirus* vaccines have been approved in Europe, Russia, and China without efficacy data in humans [12,13,14].

The regulatory strategy for ERVEBO^TM^ focused on obtaining approval in the African countries at the highest risk for future EVD outbreaks as quickly as possible. To facilitate the approval process, the WHO worked with the EMA, the African Vaccine Regulatory Forum (AVAREF), and MSD to develop a roadmap for a collaborative review procedure for the licensing and introduction of ERVEBO^TM^ in African countries [15]. This resulted in the prequalification of the vaccine by the WHO just 36 h after EMA approval, with several African countries approving the vaccine within 90 days after EMA approval (as compared with the typical one- to two-year procedure) [16].

Vaccine development involves nonclinical and clinical studies to evaluate the safety and efficacy of the product in parallel with manufacturing development, followed by submitting data supporting the product quality, safety, and efficacy to regulatory agencies for license approval and then maintaining the product post-licensure [17]. The responsibility for assuring the quality, safety, and efficacy of the products manufactured and distributed lies with vaccine manufacturers, while the National Regulatory Authorities (NRAs) have the legal authority of enforcement to ensure product quality, safety, and efficacy within their jurisdiction. 

Vaccine manufacturing is a complex process involving development to scale up the manufacturing process and to establish validated assays to test commercial products. For pandemic vaccines, in order to rapidly move forward into clinical trials and commercialization, there is little time to optimize the manufacturing process before ranges for filling must be finalized. 

Availability of process validation data is a requirement to file a vaccine with a regulatory agency. Process validation means that vaccine manufacturing must be carried out at least three times using validated equipment and under the established procedures to meet all acceptance criteria. The acceptance criteria for vaccines typically include sterility, general safety, purity, identity, and potency. Regulatory agencies sometimes repeat these tests as part of the approval process and/or for marketed products after approval. 

Non-clinical safety and efficacy studies are performed in appropriate animal models for the vaccine candidate. Repeat-dose toxicology studies for vaccines are usually performed prior to the start of human trials in a single species (for example rodents or rabbits) and include antemortem parameters and post-mortem evaluations of a comprehensive list of tissues. Additional toxicology studies, such as developmental and reproductive toxicity are performed later in development. For pandemic vaccines, it is possible to use pre-existing data supporting the vaccine platform to initiate clinical trials. For the rVSVΔG-ZEBOV-GP program, data from repeat-dose toxicology studies were not available when clinical development started in 2014. However, antemortem parameters were evaluated in NHP efficacy trials and safety data from other similar viral vectors (such as a vesicular stomatitis virus (VSV)-based HIV vaccine) [18] to support the start of clinical trials, and toxicology studies were performed in parallel with clinical development. In addition, the Public Health Agency of Canada and their partners published efficacy studies in NHPs with rVSVΔG-ZEBOV-GP that were used to support the start of clinical trials [19,20,21,22,23], and additional immunogenicity and efficacy studies in NHPs were performed in parallel with Phase 1 clinical trials to validate the selection of the vaccine dose used in Phase 2 and 3 clinical efficacy trials. 

In the development of pandemic vaccines, the phases of clinical development, such as Phase 1 and 2 safety and immunogenicity evaluation of a range of vaccine doses followed by randomized, placebo-controlled, Phase 3 trials evaluating the safety, immunogenicity, and efficacy need to be accelerated. The challenge with developing vaccines for emerging infectious diseases such as EVD is that they do not easily lend themselves to evaluations in randomized placebo-controlled trials because of low case counts in the population in the absence of an outbreak and the lack of predictability of outbreaks. The Ebola Ҫa Suffit trial conducted by the WHO in Guinea used a novel ring vaccination, cluster-randomized design that was modeled after smallpox eradication efforts to target populations at the highest risk of EBOV infection [24].

Many countries provide alternative regulatory pathways to allow the development of vaccines and therapeutics for life-threatening diseases and conditions for which no approved products are available. In the U.S.A., the traditional approval pathway requires demonstration of efficacy in clinical trials; however, alternative approval pathways can be used in the absence of data to support the traditional pathway. Accelerated approval may be based on a surrogate endpoint (e.g., immunogenicity in the case of a vaccine) or an intermediate clinical endpoint with a commitment to demonstrate efficacy post-licensure. The Animal Rule is an alternative pathway that may be used, and approval is based on evidence of effectiveness derived from appropriate studies in animals without adequate and well-controlled efficacy trials in humans. Products approved under the Animal Rule are subject to requirements for post-licensure studies to demonstrate clinical efficacy if such studies become feasible [25]. The EMA also provides alternative pathways to pursue a full marketing authorization approval when clinical efficacy data are limited, including conditional and exceptional approval pathways [26]. These pathways include post-approval requirements to perform effectiveness studies during future outbreaks. 

As a prerequisite for approval of a new vaccine, safety must be evaluated in clinical trials regardless of the ability to collect efficacy data and the pathway used to demonstrate efficacy. For most new vaccines, there are several thousand vaccinated participants in a safety database at the time of submission of the Marketing Authorization Application (MAA); however, the size of the clinical safety database for a new vaccine varies depending on its indication and whether an adverse effect was identified in clinical trials that require evaluation in a population of a specific size. 

Finally, lot-to-lot manufacturing consistency is often demonstrated using clinical immunogenicity data. In the rVSVΔG-ZEBOV-GP program, consistency of the manufacturing process was assessed through the conduct of a clinical trial that demonstrated the equivalence, in terms of immunogenicity, of three different lots of the investigational vaccine.

In order to quickly initiate clinical trials in the middle of the 2013–2016 EVD outbreak, MSD and its multiple partners worked closely with regulatory agencies in several countries to rapidly submit available data for the agencies to review and approve. Regulatory agencies also partnered to review clinical trial applications, and the AVAREF supported the joint review of clinical trial applications in western African countries. 

MSD worked closely with several regulatory agencies to ensure that the overall development plan would support vaccine licensure, which facilitated the acceleration of the rVSVΔG-ZEBOV-GP program. FDA and EMA have regulatory designations that may enhance support for medicines and vaccines targeting an unmet medical need for life-threatening diseases, including fast-track or breakthrough therapy designation in the US and priority medicines status in the European Union. MSD applied for regulatory designations to expedite the development of rVSVΔG-ZEBOV-GP based on an interim efficacy analysis of the Phase 3 Guinea Ring Vaccination Trial in which 100% efficacy was demonstrated [24]; and in June 2016, rVSVΔG-ZEBOV-GP was granted breakthrough therapy designation by the FDA and priority medicines designation by the EMA. This enabled consistent interactions with both the FDA and EMA on product development and alignment on processes and timelines prior to filing. In addition, several meetings were held with the WHO’s prequalification team to align on the process for prequalification. The WHO’s prequalification team performed a joint review of the MAA with the EMA, which facilitated rapid prequalification of the vaccine. 

In order to facilitate access to the EMA-approved vaccine in African countries, representatives from each of the participating countries participated in a collaborative review with the EMA, which was facilitated by the WHO and AVAREF organizations. AVAREF was initially created by WHO in 2006 to improve regulatory oversight of interventional clinical trials being conducted in Africa. AVAREF has since expanded its activities to include support for regional registration, and the collaborative review roadmap that was created for ERVEBO^TM^ approval can be applied to other vaccine programs in the future. 

If the product is considered of major interest to public health and addresses an unmet need, it is possible to apply for a faster review, called Priority Review, of the marketing application at the filing stage. This enables review in 8 months versus the normal 12 months by the FDA. The ERVEBO^TM^ Biologics License Application was granted Priority Review by FDA and their review was completed in 5 months, with approval obtained in December 2019. Similarly, EMA granted Accelerated Assessment for the ERVEBO^TM^ MAA in March 2019, which took 6 months instead of the standard 12 months and rendered a positive opinion for a conditional Marketing Authorization in October 2019, which was confirmed by the European Commission on 11 November 2019. ERVEBO^TM^ was prequalified the next day, on 12 November 2019, based on the parallel review performed by WHO. Significant resources are required on the part of both the manufacturer and the regulatory agencies during these rapid reviews, including review of the dossier that is typically composed of thousands of pages by the NRA, inspection of the manufacturing facility, key sites, and sponsor facilities, and potentially an independent evaluation of key test methods used to release the vaccine. The manufacturer must rapidly respond to questions asked by the NRA during the review of the MAA, which may number in the hundreds, covering the manufacturing, non-clinical, and clinical data. MAA approval occurs after all the available data have been reviewed and the vaccine has met prescribed requirements for quality, safety, and efficacy.

## 3. Manufacturing

Manufacturing process development for rVSVΔG-ZEBOV-GP was completed in the midst of outbreaks. Therefore, it was important to ensure adequate reserves of vaccine supply were available for compassionate use and expanded access clinical trial protocols, which allowed access to the vaccine prior to licensure, and in parallel to support process validation activities required for approval in the shortest time possible. 

The development of a manufacturing process to support licensure of rVSVΔG-ZEBOV-GP was completed in three stages. In the first stage, a small-scale process suitable for the manufacture of clinical trial material was implemented to produce doses at a contract manufacturing organization. This smaller clinical-scale process was used to supply material to Phase 1 clinical studies through the Ring Vaccination Trial conducted in Guinea during the 2013–2016 outbreak [24]. In the second stage, the clinical-scale process was transferred to an MSD internal clinical production facility and scaled-up to produce a larger number of doses. The process transfer and scale-up allowed MSD to replenish the limited supply of compassionate use doses to ensure an adequate level of public health preparedness in the event of future outbreaks. Scale-up of the clinical-scale process was required to develop a process capable of meeting future global demand. Lastly, as the final manufacturing facility was not yet complete, carrying out production activities in a separate clinical facility allowed accelerated outbreak support to proceed in parallel with efforts to gain licensure of the final manufacturing facility and process. In the final stage, the scaled-up process was transferred to the final manufacturing facility. 

During this final transfer, the number and extent of process changes were minimized to only those necessary to ensure the robustness of the final production process and compatibility with the production facility equipment and layout. Internal and external options were considered in selecting a manufacturing facility to support the final production of rVSVΔG-ZEBOV-GP. There were limited external options available with expertise in live virus vaccine production, experience in large-scale commercial production after regulatory approval (where requirements are typically more stringent compared with the clinical development phase), and open production capacity. Ultimately, an internal facility was selected and offered advantages for speed, capacity, and control to facilitate the rapid initial licensure of rVSVΔG-ZEBOV-GP. 

To accelerate the standard paradigm for developing a new vaccine manufacturing process, several strategic decisions were made early in the rVSVΔG-ZEBOV-GP project to shorten development time. In some cases, process technologies that supported earlier licensure were chosen in lieu of options that were more advanced but required more time to develop. Key manufacturing process parameters such as the cell line, virus strain, drug substance production platform, and drug product platform were maintained identical or as close as possible to the clinical scale process, reducing the time required to lock in a process for regulatory submission. In a traditional development paradigm, the initial clinical process might undergo further development to optimize the process with these changes requiring additional clinical studies. For rVSVΔG-ZEBOV-GP drug substance (i.e., bulk vaccine), a roller bottle production system that was used to produce early clinical material, which was more manual but well-characterized, was maintained and the number of bottles scaled up to ensure adequate capacity. For drug products (i.e., vaccine filled into single- or multi-dose vials), the frozen formulation used throughout clinical development, including efficacy studies, was maintained. Although lyophilized or liquid formulations, in principle, offer benefits for cold chain storage, the development of a new formulation requires significant efforts and there are often technical challenges with developing thermostable formulations for viral vaccines. Furthermore, to show equivalency of any new formulation to the existing frozen formulation would require additional preclinical and clinical testing. Ultimately, the frozen formulation supported the short-term goal to achieve licensure as quickly as possible but represented a tradeoff for the longer-term benefits of a lyophilized or liquid formulation that would not require long-term storage at −70 °C. 

Rather than making changes to critical product quality attributes, manufacturing process development efforts were streamlined and focused only on defining and characterizing the parameter ranges necessary to support licensure and ensure consistency of future commercial production. The quality by design approach was used to prioritize and select process parameters for development work. In addition, qualified lab-scale models in combination with statistically designed experiments were used to reduce the time to generate data to support regulatory filings and on potential interactions between process parameters. 

To enable process transfer into the final manufacturing facility as soon as it was available, manufacturing process development studies were conducted in research labs in parallel with other activities, such as final manufacturing facility readiness and analytical method development, and were completed efficiently with the end goal of preparing the regulatory filings in mind. Single-use technologies were also leveraged throughout the rVSVΔG-ZEBOV-GP process to reduce timelines for the start-up of the final manufacturing facility. 

The test methods for the release of rVSVΔG-ZEBOV-GP were discussed with the FDA and EMA prior to the start of validation to ensure that adequate testing was in place to support the preparation of an application for regulatory approval and commercial specifications upon approval. In these discussions, there was a sense of urgency to implement test methods in the shortest time possible, without compromising quality. The evaluation, selection, and transfer of test methods were completed with scientific rigor to avoid risks for the patient and to minimize the potential for supply interruptions of a vaccine critical to containing outbreaks.

Several principles were considered when selecting the test methods and testing sites for rVSVΔG-ZEBOV-GP. Internal testing sites with options to build capability or contracts with external organizations were considered, and external organizations were identified with expertise and appropriate capacity. For rVSVΔG-ZEBOV-GP, a mix of external and internal capacity was leveraged to establish analytical methods in an accelerated manner.

The production facility designed for licensed rVSVΔG-ZEBOV-GP doses includes capabilities for the manufacturing of bulk drug substances, manufacturing of drug products, labeling, and packaging operations in a single location. The proximity of manufacturing areas for each of the key steps of the production process increases the level of responsiveness to changes in production needs and enables rapid resolution of issues that can occur during the completion of a batch of a vaccine compared with facilities that are geographically separated. 

The rVSVΔG-ZEBOV-GP-dedicated production facility was created through retrofit and expansion of an existing facility to generate space for all the required process steps. This approach bypassed the lengthy timelines for the construction of a new facility. To further accelerate timelines for facility start-up, most media required for the production process was sourced as a ready-to-use liquid. Only a limited number of media components known to degrade are added to the sourced media in the facility, which reduces space requirements and complexity of production operations. In addition, orders for equipment and components were placed before the process was finalized to mitigate the challenge of long lead times. 

The validation of a vaccine production process requires the production of a sequence of batches (typically three) that conform to all process parameter ranges and analytical specifications to be registered for the product during process performance qualification (PPQ). These PPQ batches are designed to demonstrate the ability to consistently manufacture separate lots of the vaccine within specifications. Both the process and the analytical test methods are validated at the sites to be used for commercial production and quality control testing. Analytical test methods need to be qualified for the testing of any good manufacturing practice (GMP) material during the early stages of the vaccine development and manufacturing program and then validated prior to testing of commercial batches. 

Prior to the start of process validation and PPQ batches, the final manufacturing facility, including utilities and systems, and process equipment need to be qualified. To accelerate the completion of process validation requirements for rVSVΔG-ZEBOV-GP, several activities typically completed sequentially were completed in an overlapping manner. Implementation of this approach required careful coordination across the construction, qualification, process, and analytical teams, but allowed process and analytical validation timelines to be significantly compressed.

**Figure 2:**
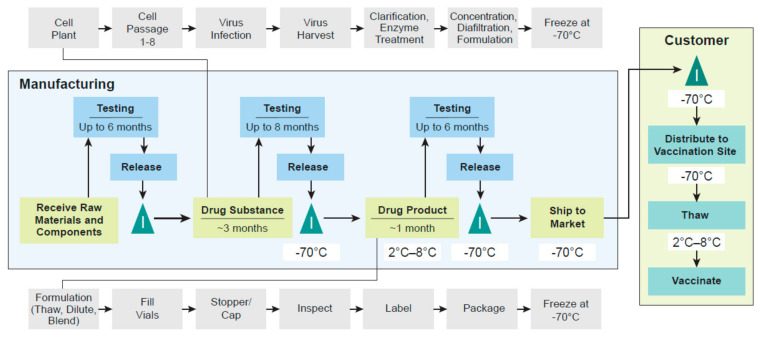
End-to-end steps for vaccine manufacturing and supply. The manufacture of a vaccine requires the completion of a sequence of activities starting from the receipt of raw materials and components through the release of a dose that is ready to be administered to a patient. At different steps of the process, inventory (I) may be held until the completion of the preceding step.

The entire process required to manufacture, test, and supply doses of rVSVΔG-ZEBOV-GP can take over a year and includes significant complexity, with testing and specifications that need to be met at multiple points throughout the process (Figure 2). The shipment of large quantities of a complex, frozen product requires careful coordination of delivery to regions that may not have consistent and adequate cold-chain capabilities. Although there are opportunities to hold inventory across the manufacturing process within allowable material expiries and vaccine shelf life or to fill drug product doses based on partial testing results, the lead times for the production of a vaccine, or any complex product, such as rVSVΔG-ZEBOV-GP are not small. This presents a challenge because of the timing and magnitude of outbreaks, and therefore, demand for the vaccine is less certain. One way to address these challenges to supply and ensure public health preparedness is to generate a stockpile of vaccine doses and to replenish the stockpile on a routine basis as doses expire. The supply of a critical vaccine such as rVSVΔG-ZEBOV-GP requires close collaboration with agencies and governments involved in addressing public health emergencies to ensure that doses are delivered equitably when and where they are needed most. During the outbreaks in the DRC, much of the available rVSVΔG-ZEBOV-GP inventory was exhausted, so MSD and the US government worked closely to ensure additional surge capacity. Additionally, MSD committed to supplying doses to the US government and the Global Alliance Vaccine Initiative (GAVI)/United Nations International Children’s Emergency Fund (UNICEF) consortium for future global needs. 

## 4. Non-Clinical Overview 

As with most vaccine discovery and development programs, the assessment of rVSVΔG-ZEBOV-GP began with non-clinical studies conducted in appropriate animal models using non-GMP research-grade vaccine material. rVSVΔG-ZEBOV-GP was initially demonstrated to be efficacious against challenge with a mouse-adapted EBOV strain in a mouse challenge model at the National Microbiology Laboratory of the Public Health Authority of Canada [27]. However, macaques are the animal model of choice for evaluating immunogenicity and efficacy of EBOV vaccines (Table 1) [28,29] because EBOV infection in cynomolgus and rhesus macaques is similar to infection in humans in terms of disease symptoms, day of onset, clinical measurements, and outcome; and NHPs are susceptible to lethal infection with wild-type strains of EBOV. 

Prior to the initiation of rVSVΔG-ZEBOV-GP Phase 1 studies during the 2013–2016 outbreak, several NHP studies were conducted with non-GMP research-grade rVSVΔG-ZEBOV-GP in a stringent NHP model for vaccine efficacy, where a 1000 plaque-forming units (pfu) intramuscular (IM) challenge dose resulted in 100% lethality in unvaccinated cynomolgus macaques [19,20,21,22,23]. In cynomolgus macaques, a single inoculation of research-grade vaccine (at ~1 × 10^7^ pfu) given by different routes (IM, oral, intranasal) was shown to protect against illness, viremia, and death after challenge with a high dose of EBOV (1000 pfu, generally thought to represent 100–1000 times the lethal dose [median lethal dose; LD_50_] in experimental animal studies). Non-immunized controls all developed illness, high viremia, and succumbed to infection within 10 days of the challenge. These studies demonstrated that a single immunization with rVSVΔG-ZEBOV-GP is highly immunogenic and effective in protection against EVD and death in the NHP model [19,20,21,22,23]. 

Despite the strong basis provided by the published NHP studies supporting advancement to clinical trials in 2014, it was unknown whether similar levels of immunogenicity or efficacy would be obtained in humans prior to initiation of clinical trials. Additionally, it was unknown whether clinical trials conducted during an unpredictable EVD outbreak would be able to attain sufficient EVD cases to demonstrate efficacy. These unknowns were shared for all vaccines under development; and a workshop was convened in December 2014, co-sponsored by the FDA, the National Institutes of Allergy and Infectious Diseases, the US Department of Defense, the Centers for Disease Control and Prevention (CDC), and the Biomedical Advanced Research and Development Authority, to discuss EBOV immunology in the context of vaccine development, clinical evaluation, and regulatory decision-making [31]. Without data demonstrating the correlates of protection for any of the vaccines, binding and neutralizing antibody assays and other functional antibody and cell-mediated immunity assays were proposed for development in both NHP studies and clinical trials to compare immune responses across species. If efficacy could not be demonstrated in late-phase clinical trials, these data would be especially important, enabling the pursuit of the Accelerated Approval Pathway or Animal Rule for licensure. 

Both the Accelerated Approval Pathway and the Animal Rule Pathway require additional NHP studies targeted at identifying immune correlates of protection and bridging those immune responses between NHPs and humans. Although the leading vaccine candidates at the time had been shown to be highly effective in animal studies, an immune correlate had not been identified for any of them, and different vaccine platforms may have different correlates of protection in addition to different thresholds [32]. Despite these potential differences between vaccine platforms and responses, it was agreed that standardized assessment of immunogenicity of the vaccines was needed as part of a global health response for EVD through a collaborative effort among government organizations and the vaccine industry. The Filovirus Animal Non-Clinical Group (FANG), co-led by a US government interdepartmental and interagency group assisted the collaboration effort, with the Medical Countermeasure Systems (MCS)-Joint Vaccine Acquisition Program (JVAP) leading the enzyme-linked immunosorbent assay development and validation effort. The group facilitated the advanced development of filovirus medical countermeasures, including standardized assays, reagents, and animal models. 

To address the urgent need for medical countermeasures against EVD, the scientific community worked together to optimize and implement the standardized FANG enzyme-linked immunosorbent assay ELISA [33,34]. Testing human and NHP samples in the same optimized and validated assay was considered critical to enabling better bridging between NHP and human immune responses. MSD worked closely with MCS-JVAP and other partners (including The United States Army Medical Research Institute of Infectious Diseases, Battelle Memorial Institute, National Institutes of Allergy and Infectious Diseases, and several vaccine developers) to assess the human ELISA reagents for detection of antibodies against EBOV GP in NHP samples using the same assay method as used for human samples. A single assay was implemented for both human and NHP samples once the FANG human ELISA was shown to have comparable assay performance for detection of NHP anti-GP antibodies. The validation process for the assay with human samples, conducted at two contract research organizations, and a subsequent validation to assess the performance of the assay with NHP samples, took nearly two years to complete [35,36]. It provides an excellent tool for bridging NHP and human responses to the same vaccine and allows for comparison of responses across studies and vaccine platforms. 

The development and validation of the EBOV neutralizing assay for rVSVΔG-ZEBOV-GP followed a different paradigm than the ELISA. In order to avoid the need to work in a biosafety level (BSL)-4 laboratory, a BSL-2 plaque reduction neutralization test (PRNT) assay, using the rVSVΔG-ZEBOV-GP virus as the target virus for neutralization was developed and validated [34]. The rVSVΔG-ZEBOV-GP virus serves as a surrogate virus for neutralization because it expresses EBOV GP surface protein similar to that of wild-type EBOV. Like the glycoprotein-enzyme-linked immunosorbent assay (GP-ELISA), the PRNT assay, which is species agnostic, was also validated for human and NHP samples to allow the bridging of immune responses between the two species. 

Repeat-dose toxicity studies in mice and NHPs and a developmental and reproductive toxicity study in rats were conducted in parallel with clinical trials and demonstrated no evidence of systemic toxicity and no effect on mating, fertility, or fetal development. A biodistribution study performed in NHPs demonstrated viral presence in multiple organs over the first few days following vaccination and localization of rVSVΔG-ZEBOV-GP at later timepoints mostly within lymphoid tissues with viral RNA detection after day 7 confined to tissues lacking potential for shedding. There was no evidence of viral distribution to the brain or spinal cord. A NHP neurovirulence study with rVSV∆G-ZEBOV-GP vaccine showed no clinical signs of significant histopathological lesions following intrathalamic administration of the vaccine [37]. 

At the start of the EVD outbreak in 2013, several pharmacology studies demonstrating robust immunogenicity and pre- and post-exposure prophylactic efficacy in mice and NHPs had already been successfully completed; however, non-clinical safety studies had not yet been conducted. Although non-clinical efficacy studies generally lack histopathology endpoints, some general safety information can be gathered via review of available study data, including physical signs or any available blood, serum, or urine sample data collected from unchallenged vaccinated control animals on the studies. In the face of an ongoing EVD outbreak, this robust, non-clinical pharmacology dataset, coupled with safety data from a similar viral vector (the VSV-based HIV vaccine) [38], and a published neurovirulence study in NHPs [37,39] were considered sufficient to enable human clinical trials to proceed. 

Emergency deployment provided a unique opportunity to gather and utilize emerging data from ongoing clinical trials and apply them prospectively to non-clinical study designs. Specifically, extensive assessment of forelimb and hindlimb joints and joint fluid was incorporated into the non-clinical toxicology and biodistribution studies based on the relatively common adverse events of arthralgia and arthritis reported in the ongoing clinical trials, revealing a transient presence of vaccine virus at relatively high titers in NHP joints that were not associated with histomorphologic changes (data on file, Merck & Co., Inc., Kenilworth, NJ, USA). Study planning also included the thoughtful selection of time points for assessment of vaccine viremia and viruria in the non-clinical safety studies, using clinical and non-clinical data to inform the study design of biodistribution and livestock transmission studies (data on file, Merck & Co., Inc., Kenilworth, NJ, USA). 

Since VSV is known to be neurovirulent, a dedicated non-clinical study for the assessment of potential neurovirulence was required. Data supporting rVSVΔG-ZEBOV-GP were available from an exploratory assessment of neurovirulence published by the New England Primate Research Center [37,39] that demonstrated a lack of neurovirulence in NHPs and provided reassurance regarding central nervous system safety prior to the initiation of rVSVΔG-ZEBOV-GP clinical trials. 

Mice have been less commonly used as the toxicology species for regulatory studies than rabbits or rats, mainly due to their small size [40]. However, the pre-existing data and experience with mouse as a model in rVSVΔG-ZEBOV-GP non-clinical pharmacology and efficacy studies made the mouse a logical choice for non-clinical safety testing [27]. Mice that were given rVSVΔG-ZEBOV-GP on days 1 and 14 showed no evidence of systemic toxicity after receiving an approximately 3030-fold excess based on pfu/kg body weight compared with the Phase 3 clinical dose and demonstrated acceptable local tolerability at the IM injection sites. A repeat dose toxicity study in NHPs was also conducted in support of rVSVΔG-ZEBOV-GP. Like the mouse toxicity study, NHPs showed no evidence of systemic toxicity after receiving an approximately 100-fold excess dose based on pfu/kg body weight compared with the Phase 3 clinical dose, and the local injection-site response was acceptable (data on file, Merck & Co., Inc., Kenilworth, NJ, USA). 

A developmental and reproductive toxicity study in pregnant female rats was conducted in parallel with Phase 3 clinical trials to evaluate embryofetal development and pre- and post-natal development. Rats received four doses, representing a 533-fold excess based on pfu/kg body weight compared with the Phase 3 clinical dose. An additional group of female rats received a single dose to evaluate toxicity resulting from a viremia response. There was no effect of rVSVΔG-ZEBOV-GP on mating, fertility, or fetal development. Viremic infection was induced during gestation, and administration of rVSVΔG-ZEBOV-GP showed no effect on mating, fertility, or fetal development following either single or multiple doses (data on file, Merck & Co., Inc., Kenilworth, NJ, USA).

To assess biodistribution, cynomolgus macaques were administered a single injection of rVSVΔG-ZEBOV-GP at 2.4 × 10^8^ pfu by the IM route. The vaccine was well tolerated, and no findings were observed at each scheduled necropsy. Vaccine viral RNA persisted primarily in lymphoid tissues by quantitative reverse transcriptase–polymerase chain reaction throughout the duration of the study (112 days) (data on file, Merck & Co., Inc., Kenilworth, NJ, USA). A subsequent plaque assay detected replication-competent virus limited to day 1 post-vaccination. There was no evidence of viral replication at any other time point measured (days 56, 84, and 112). Viral RNA after day 7 was generally confined to tissues lacking potential for shedding in excretions or secretions and showed no evidence of distribution to the brain or spinal cord at any time point. rVSVΔG-ZEBOV-GP viral RNA persisted in lymphoid tissues, but there was no evidence for persistence of infectious virus by plaque assay (data on file, Merck & Co., Inc., Kenilworth, NJ, USA). 

## 5. Clinical Development

Prior to the 2013–2016 EVD outbreak in western Africa, rVSVΔG-ZEBOV-GP had not been evaluated in humans. Multiple organizations including the WHO, the CDC, Médecins Sans Frontières, the National Institutes of Health, the Biomedical Advanced Research and Development Authority, the Walter Reed Army Institute of Research, the MCS-JVAP, Public Health Authority of Canada, the Defense Threat Reduction Agency, NewLink Genetics Corporation (Bio-Protection Systems Corporation, AMES, IA, USA), in addition to numerous universities and ministries of health were involved in responding to the unprecedented 2013–2016 EVD outbreak. MSD was approached to participate in the rVSVΔG-ZEBOV-GP development program in late 2014 and accepted the responsibility to lead this collaborative partnership with the aim of bringing a vaccine to licensure as quickly as possible. 

The clinical trials included in the initial MAA were conducted in 10 countries in North America, Europe, and Africa, permitting an evaluation of the vaccine in geographically and demographically diverse populations (Table 2). Four additional trials (V920-013, 0-014, V920-015, and V920-016) were ongoing at the time of writing this review to assess the vaccine in participants at occupational risk for EVD (e.g., BSL-4 lab workers and deployed health care workers), HIV-positive participants, and children. The V920-013 trial, conducted in the US, is assessing pre-exposure prophylaxis in people at potential occupational risk for exposure to EVD, and the V920-014 trial is being conducted in Gabon to assess safety, tolerability, and immunogenicity in children 1–12 years old, in addition to transmission to household contacts. The V920-015 and V920-016 clinical trials expanded enrollment into three new African countries. Expanded access protocols were utilized to provide access to rVSVΔG-ZEBOV-GP in Guinea, Sierra Leone, and Liberia during the final phases of the 2013–2016 EVD outbreak [41,42]. Two new EVD outbreaks that began in 2018 also utilized expanded access protocols to vaccinate a significant number of participants (>300,000) in the DRC [43] and surrounding countries. 

Data from over 15,000 adult participants vaccinated with rVSVΔG-ZEBOV-GP in clinical trials were presented in the MAA. The majority of participants (and all participants in Phase 2/3 trials) were vaccinated with the nominal dose of rVSVΔG-ZEBOV-GP ≥2 × 10^7^ pfu. The clinical trials mostly enrolled and vaccinated HIV-negative adults 18 to 65 years of age. However, approximately 530 adults ≥65 years of age, 230 children 6 to 17 years of age, 270 women who subsequently became pregnant, and 20 HIV-positive individuals were also vaccinated with rVSVΔG-ZEBOV-GP in the first 12 clinical trials, and safety was evaluated in all the clinical trials. 

Due to the rapidly advancing EVD outbreak in western Africa in 2014, a decision on the potency of a vaccine dose was made as soon as the initial Phase 1 clinical trial results became available. This was based on safety, immunogenicity, and that animal challenge studies were expected to be efficacious in humans. Instead of studying this dose potency in Phase 2 proof of concept trials (as in a typical development program), the selected dose was used in Phase 2/3 clinical trials in the countries affected by the outbreak. 

Data from all relevant clinical trials (e.g., with a final formulation and at the final dose) should be summarized to comprehensively assess safety, and if possible, these data should be integrated with the MAA to facilitate the presentation of a single point estimate for the frequency of adverse reactions and to optimize the ability to look for rare serious adverse events [54]. Variations in the trial designs throughout the rVSVΔG-ZEBOV-GP clinical development program (e.g., open-label versus blinded, utilizing or not utilizing memory aids, varying durations, different CSR MedDRA version) were a major limiting factor in the ability to integrate safety data (Table 2). Differences in geographical regions (e.g., Africa only versus Europe/North America only), outbreak and trial setting circumstances (e.g., outbreak versus non-outbreak, community versus clinical settings), and methods for data collection (e.g., phone contact with participants versus in-person visits) were also limitations to safety data integration. Of note, the trials were not designed or conducted with the final objective of data integration, but ultimately, it was feasible to integrate serious adverse event (SAE) data from blinded trials because of the more uniform detection methods for SAEs across trials (Table 2). The discrepancies in the safety data collection methods represent a drawback to conducting many trials sponsored by different parties in parallel. The advantage was the speed to completion of the clinical trial program. If 12 clinical trials with standardized safety data collection methods were conducted by a single sponsor, it could have resulted in the clinical development program lasting 5–10 years longer due to resource constraints, rather than the approximate four years in which the rVSVΔG-ZEBOV-GP clinical program was conducted. These tradeoffs must be carefully weighed and considered when developing a vaccine for pathogens with pandemic potential.

The safety data suggest that rVSVΔG-ZEBOV-GP has an acceptable safety profile in healthy, non-pregnant adults 18 years and older. African and non-African Phase 2/3 placebo-controlled trials using the final dose informed the United States prescribing information and the European Union Core Safety Profile [50,51,52]. The most common injection-site adverse events were injection site pain (70%), swelling (17%), and redness (12%) and were generally mild-to-moderate in intensity and short in duration. The most commonly reported systemic adverse events following vaccination with rVSVΔG-ZEBOV-GP were headache (37%), feverishness (34%), muscle pain (33%), fatigue (19%), joint pain (18%), nausea (8%), arthritis (5%), rash (4%), and abnormal sweating (3%). 

Overall, arthralgia of mild to moderate intensity, generally reported in the first few days following vaccination and resolving within one week after onset, was reported in 7% to 40% of vaccine recipients in blinded, placebo-controlled studies. Severe arthralgia, defined as preventing daily activity, was reported in up to 3% of participants overall. The majority of joint-related adverse events were reported in the first few weeks after vaccination, were mild-to-moderate in intensity, and were resolved within days to weeks. However, a few participants reported prolonged joint symptoms (up to two years) [55].

All but one blinded, placebo-controlled study reported rash in <9% of participants after administration of rVSVΔG-ZEBOV-GP. In a Phase 1/2 study conducted in Switzerland (NCT02287480), rash was reported in four (25%) rVSVΔG-ZEBOV-GP recipients and one (7.7%) placebo recipient. White blood cell counts were assessed in 697 rVSVΔG-ZEBOV-GP recipients. Decreases in lymphocytes were reported in up to 85% of vaccinees and decreases in neutrophils were reported in up to 43% of vaccinees, but no associated infections were reported [55]. 

There were few vaccine-related SAEs reported. Among the 15,399 rVSVΔG-ZEBOV-GP recipients included in the safety assessment of the MAA, two serious adverse reactions each of pyrexia and anaphylaxis were reported as vaccine-related. None of these serious adverse reactions were fatal [55]. 

**Figure 3:**
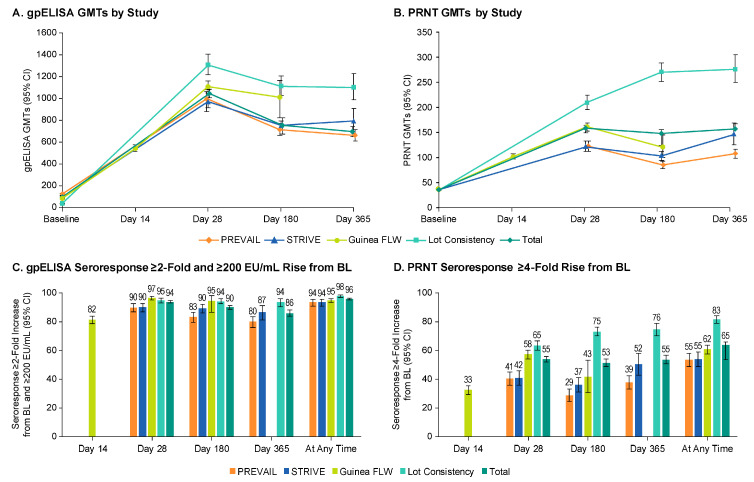
Glycoprotein-enzyme-linked immunosorbent assay (GP-ELISA), plaque reduction neutralization test (PRNT), and seroresponse results by study for Partnership for Research on Ebola Virus in Liberia (PREVAIL), Sierra Leone Trial to Introduce a Vaccine against Ebola (STRIVE), Guinea Front Line Worker (FLW) trial study, lot consistency study, and integrated results. (**A**): GP-ELISA GMTs (**B**): PRNT GMTs (**C**): GP-ELISA seroresponse ≥2-fold and ≥200 EU/mL rise from baseline (D): PRNT seroresponse ≥4-fold rise from baseline.

The immunogenicity data collected in the rVSVΔG-ZEBOV-GP program were also variable. The primary difference was the use of non-validated local assays in Phase 1, whereas validated assays were conducted at a central laboratory in Phase 2 and 3 trials. Ultimately, all partners used a collaborative approach to facilitate the use of validated immunogenicity testing in a binding GP-ELISA and functional neutralizing PRNT in the Partnership for Research on Ebola Virus in Liberia (PREVAIL), Sierra Leone Trial to Introduce a Vaccine against Ebola (STRIVE), Guinea Front Line Worker (FLW), and lot consistency clinical trials, which allowed for the assessment and integration of immunogenicity data from the Phase 2 and 3 clinical trials in Liberia, Sierra Leone, and Guinea, the three countries most impacted by the 2013–2016 EVD outbreak (Figure 3 and Table 3). 

The PREVAIL trial, conducted in Liberia, was a Phase 2, double-blind, randomized trial comparing two vaccine candidates—rVSVΔG-ZEBOV-GP and the ChAd3-EBO-Z (GlaxoSmithKline)—with placebo [50]. The median age of participants was 30 years old, the majority were male (63.6%), and 100% were Black. By the first month post-vaccination, 90% of rVSVΔG-ZEBOV-GP recipients developed an antibody response (based on the seroresponse definition of ≥ four-fold increase in log_10_ titer from baseline) compared with 2.8% of placebo recipients (*p* < 0.001), and at 12 months, antibody responses in rVSVΔG-ZEBOV-GP recipients (79.5%) remained significantly greater than in placebo recipients (6.8%, *p* < 0.001). 

The STRIVE study was a Phase 2/3 unblinded, uncontrolled trial that individually randomized participants (*n* = 7998) to immediate (within seven days of enrollment, *n* = 4177) or deferred (18–24 weeks after enrollment, *n* = 3821) vaccination with rVSVΔG-ZEBOV-GP [51]. An immunogenicity sub-study of STRIVE enrolled participants from the study site of the Connaught Hospital, Freetown, Sierra Leone, during the trial and followed them for 12 months (*n* = 506). More than half of the participants were men (57.9%), the median age was 32 years old, and 100% were Black. By month 1, 90% of vaccine recipients developed an antibody response (based on the seroresponse definition of ≥ two-fold increase in antibody titer from baseline and antibody titer ≥200 EU/mL) and at 12 months, 88% of vaccine recipients maintained an antibody response (Mahon et al., manuscript submitted).

The FLW trial conducted in Guinea was an open-label, non-randomized, single-arm safety and immunogenicity study of FLWs, including personnel working in Ebola or non-Ebola health facilities and services (*n* = 1118) [53]. The mean age of vaccinated participants was 34.5 years old and most were male (73.4%). At 14 days post-vaccination, 65.1% (95% CI: 62.1, 68.1) of vaccinees seroresponded. At 28 days post-vaccination, 97% (95% CI: 84.1, 88.4) of vaccinees seroresponded (based on the seroresponse definition of ≥four-fold increase from baseline), and the seroresponse persisted at 180 days post-vaccination for 90.7% (95% CI: 82.0, 95.4) of those with results (*n* = 90).

The lot consistency trial conducted in the United States, Canada, and Spain was a randomized, double-blind, placebo-controlled trial of safety and reactogenicity data in healthy adults [52]. The mean age was ~41 years old, approximately half were women (53%), and the majority were white (68%). At 28 days post-vaccination, 95.4% (95% CI, 93.6, 96.8) of recipients in the standard dose group and 98.2% (95% CI, 95.4, 99.5) of the high-dose group had seroresponded (defined as a ≥two-fold increase in antibody over baseline and antibody titer ≥200 EU/mL), and the seroresponse persisted at 24 months in >90% of vaccinees from both dose groups.

The PREVAIL [50], STRIVE [51], and Ebola Ҫa Suffit [24] trials were planned to evaluate efficacy. However, due to the waning outbreak at the time the trials started, only Ebola ça Suffit yielded enough EVD cases to demonstrate efficacy [24]. Therefore, the PREVAIL and the STRIVE clinical trials assessed safety and immunogenicity, but not efficacy. The Ebola Ҫa Suffit trial was a Phase 3, open-label, cluster-randomized ring vaccination trial conducted in Guinea (*n* = 5837) [24]. The primary objective was to assess the efficacy of rVSVΔG-ZEBOV-GP for the prevention of EVD. The median age of participants was 35 years old, and there were more men (62%) than women (38%). The preliminary results indicated 100% vaccine efficacy with a 95% CI of 74.7% to 100.0%, and the final results confirmed that vaccine efficacy was 100% (95% CI: 63.5% to 100%). There were no cases of confirmed EVD observed in the immediate vaccination clusters, and there were 10 confirmed cases of EVD observed in four delayed vaccination clusters between day 10 and day 31 post-randomization. 

The Ebola Ҫa Suffit trial, with its unique design adopted from the smallpox eradication efforts, took advantage of the efficiency of vaccinating participants around identified index cases and ended up being the only clinical trial providing evidence of efficacy for any *Ebolavirus* vaccine to date. The ring design of Ebola Ҫa Suffit maximized the declining number of EVD cases by clustering vaccination around all reported cases in real time as they were identified and then randomizing those clusters to either immediate vaccination or vaccination delayed by 21 days [24]. Simply stated, the WHO brought the trial to the cases rather than setting up clinics and waiting for those cases to occur. This unique design allowed for the evaluation of the efficacy of rVSVΔG-ZEBOV-GP at the individual participant level, in addition to an assessment of the effectiveness of ring vaccination as a containment strategy. Moreover, it demonstrated that it was possible to conduct a randomized trial during the outbreak despite substantial opposition to this type of trial by stakeholders involved in the response [56]. Since the immune responses in Liberia, Sierra Leone, and Guinea were similar, it was possible to extrapolate those results to the separate population in Guinea that was included in the Ebola Ҫa Suffit trial, in which efficacy results were obtained, but due to logistical reasons, no immunogenicity specimens were collected [24]. Although it was not possible to assess immune correlates of protection (i.e., determine which immune response in the Ebola Ҫa Suffit efficacy clinical trial was associated with protection) at the individual level, an immune correlate of protection was assessed at the population level by describing point estimates of efficacy in the Ebola Ҫa Suffit trial and point estimates of immunogenicity in integrated data from the PREVAIL, STRIVE, and FLW trials [57]. The statistical immune correlate of protection analysis was not critical for the rVSVΔG-ZEBOV-GP clinical development program because human efficacy data were available, but it may be important for future generations of EBOV vaccines. Additionally, immune correlates of protection may be important to expand the indication to special populations such as children and persons infected with HIV, in whom efficacy has not yet been assessed.

It is important to note that there were personal safety considerations in play when importing, handling, and testing immunogenicity specimens from participants in countries where the EBOV was circulating. All specimens collected in western Africa from the PREVAIL, STRIVE, and FLW trials were gamma-irradiated prior to arrival at the central laboratory in the US. This treatment enabled samples to be safely handled under BSL-2 conditions by laboratory workers analyzing these specimens. Interestingly, gamma irradiation resulted in an approximate 20% increase in non-specific binding pre-vaccination and an approximate 20% decrease in specific binding post-vaccination [58], which had the greatest impact on the assessment of fold-increase pre- to post-vaccination and must be considered when comparing immune responses of specimens that have been irradiated (PREVAIL, STRIVE, and FLW trials) versus those that have not been irradiated (e.g., lot consistency trial). Additionally, since there was no immune correlate of protection established at the time, it was important to determine a serum status cutoff that could define seropositivity and seroresponse. Non-validated GP-ELISA results from rVSVΔG-ZEBOV-GP and placebo recipients in Phase 2 PREVAIL trial conducted in Liberia were assessed to determine which serum status cutoff optimally differentiated between participants that received vaccine and placebo and therefore defined seropositivity and seroresponse [50]. Based on these results, it was determined that 200 EU/mL optimally determines seropositivity and that 200 EU/mL and a two-fold rise pre- to post-vaccination optimally determines seroresponse [59]. 

## 6. Ongoing Work and Relevance to Vaccine Development for Other Emerging Infectious Diseases

Beyond the trials that were included in the dossier to support initial licensure, a number of additional studies have been or are being conducted to expand the knowledge base for rVSVΔG-ZEBOV-GP. These include assessments of the durability of responses, the impact of booster doses, and safety and immunogenicity in special populations, including children and HIV-positive participants. In addition, expanded access/compassionate use protocols have been extensively utilized in the context of the recent Ebola outbreaks in the DRC to provide access to the vaccine for individuals at-risk for EVD, including children as young as 6 months of age and women who are pregnant (post first trimester) or lactating. Cumulatively, these studies are greatly expanding our understanding of the performance of the vaccine and provide a model for expanding the evaluation of safe and effective vaccines for emerging viruses to vulnerable populations. The studies include the following:

- FLW trial: Part B of the Ebola Ҫa Suffit trial was an open-label safety and immunogenicity trial conducted in 2115 frontline workers in Guinea. The study was sponsored by the WHO and conducted by Médecins Sans Frontieres. The study was initiated at the same time as the Ebola Ҫa Suffit trial, but since efficacy was not assessed in this study, efforts were focused on the safety and efficacy trials for the initial license submissions. Data from the trial expanding our knowledge of the safety and immunogenicity of the vaccine have recently been published [53]. Importantly, given the conduct of this trial in the same country and at the same time as the efficacy trial, population-based approaches to evaluate potential correlates of protection for ERVEBO^TM^ have been assessed using the data from the trial [57]. This evaluation suggests that the GP-ELISA assay, originally established by the FANG and subsequently validated at Q^2^ Solutions, correlates with protection for this vaccine, although a specific protective threshold was not defined. Evidence for a correlate of protection for rVSVΔG-ZEBOV-GP provides additional information for groups such as the WHO Strategic Advisory Group of Experts (SAGE) as they recommend the best way to use the vaccine to have the largest public health impact.
- PREPARE (Pre-Exposure Prophylaxis in People at Potential Occupation Risk for Ebola Virus Exposure) trial: This ongoing National Institute of Allergy and Infectious Diseases (NIAID)-sponsored, randomized, and controlled trial being conducted in the US and Canada evaluates the durability of immune responses to rVSVΔG-ZEBOV-GP in individuals who are at occupational risk of exposure to Ebola (e.g., through working in BSL-4 laboratories, etc.) and includes randomization to assess the impact of a booster dose given at 18 months.
- Partnership for Research on Ebola Vaccination (PREVAC) Trial: This large randomized, placebo-controlled trial, which is evaluating three different vaccination strategies, including one or two doses of rVSVΔG-ZEBOV-GP, in adult and pediatric participants in Guinea, Liberia, Mali, and Sierra Leone is sponsored by the National Institute of Allergy and Infectious Diseases (NIAID), Institut National de la Santé et de la Recherche Médicale (INSERM), and the London School of Hygiene and Tropical Medicine (LSHTM). The trial, which is fully enrolled, includes children as young as 1 year of age. The data for the primary endpoint at one-year post-dose 1 is expected to be released soon. Additional follow-up of participants for five years post-dose 1 is planned. This study will provide critical data on the safety, tolerability, and immunogenicity of rVSVΔG-ZEBOV-GP in pediatric populations as young as 1 year of age and is expected to serve as the basis for expanding the indication, which is currently limited to adults, to include children.
- ACHIV (African-Canadian Study of HIV-Infected Adults and a Vaccine for Ebola) trial: This randomized, placebo-controlled trial is evaluating the safety and immunogenicity of rVSVΔG-ZEBOV-GP in HIV-positive adults and adolescents and includes the evaluation of one and two doses. This trial is sponsored by the University of Dalhousie and is being conducted in Burkina Faso, Canada, and Senegal with ongoing enrollment. Given that Ebola outbreaks may overlap with areas of high HIV prevalence, understanding the safety and immunogenicity of rVSVΔG-ZEBOV-GP in these populations is very important. It is noteworthy to say that this trial has been temporarily paused due to SARS-CoV-2, highlighting the complexity of conducting clinical trials in the context of outbreaks.
- Expanded access protocols related to the Ebola outbreaks in the DRC: Working with the Ministries of Health and local researchers, WHO has implemented expanded access protocols in the DRC, Uganda, Rwanda, South Sudan, and Burundi. These protocols are designed to provide access to individuals at potential risk of EVD by virtue of being a contact or contact of contacts for a case of EVD or being a healthcare provider or FLW in a region involved in an active outbreak or in a neighboring region that is at risk of spread. These protocols have been extensively used as part of the Ebola response to the recent outbreaks in the DRC (beginning with the Equateur Province outbreak in May 2018). Through these protocols, more than 300,000 individuals have been vaccinated in the DRC, including children as young as 6 months of age and women who are pregnant (post first trimester) or lactating [60]. In addition, more than 14,000 individuals have been vaccinated in the neighboring countries. Data from these expanded access efforts are expected to provide information on safety in these important populations and to provide information on the effectiveness of the vaccine, potentially including information on the durability of protection for rVSVΔG-ZEBOV-GP. The data generated through these expanded access efforts are much closer to “real world” data and will be an important complement to the data generated through randomized controlled trials.

Through all of these efforts, our understanding of the safety, efficacy, and utility of rVSVΔG-ZEBOV-GP is being enhanced, allowing more informed recommendations on the best use of the vaccine as a tool to prevent the devastation that comes with EVD. In addition, these efforts have provided significant learnings on general approaches and hurdles for the development of vaccines for emerging infectious diseases [7] that are particularly relevant as we face the SARS-CoV-2 pandemic. With each new outbreak and each new pathogen, the public health community gains experience on what works and what does not, which hopefully translates into safer, better vaccines made available to those at-risk more quickly than ever before. 

## 7. Conclusions

In conclusion, a worldwide partnership provided the foundation upon which rVSVΔG-ZEBOV-GP could be successfully developed and licensed in approximately 5 years. A coordinated global effort was required to acquire a large safety database from multiple clinical trials with diverse populations, during which time manufacturing processes were developed, and doses were supplied to public health agencies for use in response to ongoing EVD outbreaks. The expansion of vaccine manufacturing infrastructure globally is critical for ensuring the ability of the global public health community to respond to emerging infectious disease threats as quickly as possible. In addition, efforts are ongoing to better understand the safety, immunogenicity, and utility of rVSVΔG-ZEBOV-GP in additional populations not studied in the initial clinical development program. 

## Figures and Tables

**Table 1:** Clinical manifestations in different animal models of filovirus infections [30].

Manifestation	Immuno-Competent Mouse	Immuno-Compromised Mouse	Guinea Pig	Syrian Hamster	Ferret	NHP	Human
Lymphopenia	Yes	Yes	Yes	Yes	Yes	Yes	Yes
Liver damage	Yes	Yes	Yes	Yes	Yes	Yes	Yes
Thrombocytopenia	Yes	Yes	Yes	Yes	Yes	Yes	Yes
Coagulopathy	No	Unknown	Yes	Yes	Yes	Yes	Yes
Cytokine Storm	Yes	Yes	Unknown	Yes	Unknown	Yes	Yes
Rash	No	No	No	Yes	Yes	Yes	Yes
Hemorrhage signs	No	Yes	Unknown	Yes	Yes	Yes	Yes

NHP: Non-human primates; Virus is host-adapted for mouse, guinea pig, and Syrian hamster and wild-type for ferret, NHP, and Human.

**Table 2:** Summary of clinical trial programs.

Protocol Number, Trial Name, Country Location, and Trial Registry Number	Trial Description	[Sponsor] External Trial Partner Organization/Funders, and Academic Partners	N Vaccinated with rVSVΔG-ZEBOV-GP	Dose Levels (pfu)	Subject Memory Aid Use (Y/N)	CSR MedDRA Version	AE Category				
							Solicited Injection Site and Systemic AEs	Unsolicited AEs	SAEs	Viremia and Viral Shedding	Clinical Laboratory Safety Tests
Phase 1 *											
V920-001 US NCT02269423	Randomized single-center, double-blind, placebo-controlled, dose- escalation study [44]	[BPS/NLG] Walter Reed Army Institute of Research/DTRA, Imperial College of Science, Technology, and Medicine; University of Maryland School of Medicine, University of Texas-Austin	30	3 × 10^6^, 2 × 10^7^, 1 × 10^8^ (*n* = 10 each) or Placebo (*n* = 9)	Yes	17.0	Day 1–14	Day 1–28	Day 1–180	Day 1, 3, 7, 14	Day 0, 1, 3, 7, 28, 180
V920-002 US NCT02280408	Randomized double-blind, placebo-controlled, dose- escalation study [44]	[BPS/NLG] NIH/NIAID	30	3 × 10^6^, 2 × 10^7^, 1 × 10^8^ (*n* = 10 each), Placebo (*n* = 9) ^†^ Second identical dose at D28	Yes	17.0	Day 1–14	Day 1–28	Day 1–365	Day 0, 3, 7 following each dose	Day 0, 7, 28, 35, 56 following each dose
V920-003 Canada NCT02374385	Randomized single-center, double-blind controlled, dose-ranging study [45]	[BPS/NLG] CIHR; PHAC, University of Ottawa, Dalhousie University	30	1 × 10^5^, 5 × 10^5^, 3 × 10^6^ (*n* = 10 each), Placebo (*n* = 10)	Yes	17.0	Day 1–14	Day 1–28	Day 1–180	Day 1, 3, 7, 14	Day 0, 1, 3, 7, 28, 180
V920-004 US NCT02314923	Randomized multi-center, double-blind, placebo- controlled, dose-response study [34]	[BPS/NLG] BARDA	418	3 × 10^3^, 3 × 10^4^, 3 × 10^5^ (*n* = 64 each) 3 × 10^6^ (*n* = 84) 9 × 10^6^, 2 × 10^7^ (*n* = 47 each) 1 × 10^8^ (*n* = 48) Placebo (*n* = 94)	Yes	17.0	Cohort 1: Day 1–14 Cohort 2: Day 1–56	Cohort 1: Day 1–14 Cohort 2: Day 1–56	Day 1–360	Day 0, 1, 2, 3, 4, 7, 14, 28	Day 0, 7, 28
V920-05 Switzerland NCT02287480	Dose-finding, randomized, single-center, double-blind ^†^, placebo- controlled study [46]	[University Hospitals of Geneva] WHO, Wellcome Trust, Innovative Medicines Initiative, University Hospitals of Geneva	102	3 × 10^5^ (*n* = 51) 1 × 10^7^ (*n* = 35) 5 × 10^7^ (*n* = 16) Placebo (*n* = 13)	Yes	17.0	Day 1–14	Day 1–28	Day 1–365	Day 0, 1, 3, 7	Day 0, 1, 3, 7, 14, 28, 365 (only blood count at Day 365)
V920-006 Germany NCT02283099	Open-label, dose-escalation study [47]	[Universitätsklinikum Hamburg-Eppendorf] WHO; Wellcome Trust	30	3 × 10^5^, 3 × 10^6^, 2 × 10^7^ (*n* = 10 each)	Yes	17.0	Day 1–14	Day 1–28	Day 1–180	Day 0, 1 to 7, 14, 28	Day 0, 1, 3, 7, 14, 28, 180
V920-007 Gabon PACTR201411000919191	Randomized open-label, dose-escalation study [48]	[Universitätsklinikum Tübingen] WHO; Wellcome Trust, St. George’s University of London, Medical University Vienna, Austria	115 ^‡^	3 × 10^3^ (*n* = 20) 3 × 10^4^ (*n* = 20) 3 × 10^5^ (*n* = 20) 3 × 10^6^ (*n* = 39) 2 × 10^7^ (*n* = 16)	Yes	17.0	Day 1–14	Day 1–28	Day 1–365	Day 0, 1, 2, and 7	Day 0, 1, 2, 7, 28, 84, 180, 365
V920-008 Kenya NCT02296983	Open-label, dose-escalation study [49]	[University of Oxford] WHO; Wellcome Trust	40	3 × 10^6^ 2 × 10^7^ (*n* = 20 each)	Yes	17.0	Day 1–14	Day 1–28	Day 1–365	Day 1, 3, 7	Day 0, 7, 30
V920-009 PREVAIL Liberia NCT02344407	Randomized double-blind, placebo-controlled, 3-arm trial [50]	[NIH/NIAID] Liberian Ministry of Health and Social Welfare, BARDA, GlaxoSmithKline, University of Minnesota	500	2 × 10^7^ (*n* = 500) GSK (*n* = 500) Placebo (*n* = 500)	No	20.0	Week 1, Week 2, Month 1 ^₶^	Week 1 and Month 1	Wk 1, Month 1 and 2, every 2 Months to trial end	Not collected	At Week 1 and Month 1
V920-014 Lambaréné, Gabon Not registered	Randomized, open-label, controlled	Centre de Recherches Médicales de Lambaréné (CERMEL),	Planned: 40	2 × 10^7^ *n* = 40 Varicella vaccine N = 20	No	N/A	Days 1–28	Days 1–28	Days 1–365	Days 1–56	Screening, D7, 28, 84, 180, 365
Phase 2/3											
V920-010 Ebola Ҫa Suffit Guinea PACTR201503001057193	Open-label, cluster-randomized ring vaccination trial [24]	[WHO] Norwegian Research Council; MSF; Wellcome Trust; PHAC, Guinea Ministry of Health and Public Hygiene	5837	2 × 10^7^ (*n* = 5837)	No	N/A ^§^	Minute 30, Day 3, and Day 14	Day 1–14	Day 1–84	Not collected	Not collected
V920-011 STRIVE Sierra Leone NCT02378753	Randomized unblinded trial design [51]	[US CDC] BARDA, Sierra Leone Ministry of Health and Sanitation, College of Medicine and Allied Health Sciences—Sierra Leone	7998	2 × 10^7^ (*n* = 7998)	Yes	19.0	Safety sub study participants: Day 0–28	Overall pop: Day 0–28	Overall pop: Day 0–180	Not collected	Not collected
V920-012 Lot Consistency US, Canada, Spain NCT02503202	Randomized placebo-controlled, safety and lot consistency immunogenicity study [52]	[MSD, a subsidiary of Merck & Co., Inc, Kenilworth, NJ, USA] BARDA; Dalhousie University	1061	2 × 10^7^ (*n* = 797) 1 × 10^8^ (*n* = 264) Placebo (*n* = 133)	Yes	19.1	Day 1–42 ^¥^	Day 1 to 42	Day 1 to Month 24	Not collected	As needed for arthralgia, arthritis, rash or vesicles— follow-up only
V920-013 PREPARE US, Canada NCT02788227	Randomized open-label, booster or no booster at 18 months in individuals at potential occupational risk	[NIH/NIAID] BARDA, University of Texas, Galveston, TX, Emory University, PHAC, Winnipeg; CIRN, University of Nebraska Medical Center, Boston Medical Center/Boston University, Universitätsklinikum Hamburg-Eppendorf	Planned enrollment N up to 1000	2 × 10^7^	Yes	N/A	Day 1–14, Month 1, Month 18 (booster), Month 19 (post-booster)	Day 1–42	Day 1 to Year 3	Not collected	Not collected
V920-015 ACHIV Canada, Burkina Faso, Senegal NCT03031912	Randomized double-blind, placebo- controlled, one or two doses of rVSVΔG-ZEBOV-GP	[Dalhousie University] BARDA, CIRN	Planned enrollment ~250	2 × 10^7^ pfu/mL (n~200) Placebo (n~50)	Yes	N/A	Day 1, 3, 7, 14, 28, 42	Day 1, 3, 7, 14, 28, 42	Day 0–365	Day 3, 7, 14, 28, 42	Only as clinically needed
V920-016 PREVAC Guinea, Liberia, Mali, Sierra Leone NCT02876328	Randomized double-blind, placebo- controlled trial of three vaccine strategies (Ad26.ZEBOV/MVA-BN-Filo vaccine-Janssen, rVSVΔG-ZEBOV-GP vaccine-MSD with or without boost at 56 days) in adults and children ≥1 year	[Office of Clinical Research Operations and Regulatory Compliance Division of Clinical Research NIAID, NIH; Institut National de la Santé et de la Recherche Médicale; LSHTM] BARDA, MSD, Janssen, University of Minnesota	~1822	2 × 10^7^ pfu/mL, (N~1822) Also includes Janssen vaccine and placebo	No	N/A	Adults: Day 0, 7, 14, 28, 56, 63, Month 3 Children: Day 0, daily contacts, Day 1–6, Day 7, 14, 28, 56, 63, Month 3	Grade 3 and 4 unsolicited AEs only. Adults: Day 0, 7, 14, 28, 56, 63, Month 3 Children: Day 0, daily contacts Day 1–6, Day 7, 14, 28, 56, 63, Month 3	Day 0-Month 12	Subset of children: Day 0, 7, 14, 28, 56, 63, Month 3	Adults: Day 0 Children: Day 0, 7, 63
V920-018 Front-Line Workers (FLW) Guinea PACTR201503001057193	Open-label, cluster-randomized ring vaccination trial [53]	[WHO] University of Maryland, University of Bern, LSHTM, University of Florida	2016	2 × 10^7^(N = 2016)	No	N/A	Not collected	Day 3, 14	Day 0–84	Not collected	Not collected

* All Phase I assay work was done by the United States Army Medical Research Institute of Infectious Diseases with funding from the Joint Vaccine Acquisition Program. ^†^ Participants in the V920-002 trial received two doses of rVSVΔG-ZEBOV-GP on days 0 and 28 post-vaccination. Data for participants who received the second dose are presented separately from participants who received a single dose. ^‡^ Additional 40 pediatric subjects also included (6–12 and 13–17 years of age, each *n* = 20). ^₶^ The V920-009 trial did not collect a specific adverse event (AE) onset date or stop date; all other trials collected AE onset and stop dates for solicited AEs. ^§^ Adverse events were not encoded using MedDRA for the V920-010 trial. ^¥^ Injection-site AEs were solicited from day 1 to 5 post-vaccination in the V920-012 trial; joint and skin events were solicited from day 1 to 42. No other solicited systemic AEs were collected in this trial. AE = adverse event; BARDA: Biomedical Advanced Research and Development Authority; BPS: BioProtection systems; CIRN: Canadian Immunization Research Network; CDC: Centers for Disease Control; CIHR: Canadian Institutes of Health Research; CSR: clinical study report; DTRA: Defense Threat Reduction Agency; LSHTM: London School of Hygiene and Tropical Medicine; MSF: Médecins Sans Frontières; N/A: not applicable; NIH: National Institutes of Health; NIAID: National Institute of Allergy and Infectious Diseases; NLG: NewLink Genetics Corporation; pfu: plaque-forming units; PHAC: Public Health Agency of Canada; PREVAIL: Partnership for Research on Ebola Virus in Liberia, SAE: serious adverse event; STRIVE: Sierra Leone Trial to Introduce a Vaccine Against Ebola; US: United States; WHO: World Health Organization.

**Table 3:** GP-ELISA, PRNT, and seroresponse results by study for PREVAIL, STRIVE, FLW, lot consistency study, and integrated results.

	Baseline	Day 14	Day 28	Day 180	Day 365	Day 14	Day 28	Day 180	Day 365	At Any Time
**GP-ELISA GMTs (95% CI)**						**GP-ELISA Seroresponse two-fold and 200 EU/mL** **Increase from Baseline**				
**FLW**	82.8 (79.4, 86.3)	543.2 (512.5, 575.8)	1106.5 (1053.4, 1162.2)	1008.8 (849.8, 1197.6)	--	81.5 (78.1, 83.9)	96.7 (95.3, 97.7)	94.6 (86.7, 98.5)	--	94.9 (93.4, 96.1)
**PREVAIL**	121.8 (112.1, 132.4)	--	994.7 (915.0, 1081.3)	712.2 (659.4, 769.3)	661.4 (613.2, 713.4)	--	90.0 (86.9, 92.6)	83.2 (79.5, 86.5)	80.1 (76.2, 83.7)	93.8 (91.1, 95.8)
**STRIVE**	97.1 (89.7, 105.0)	--	969.9 (885.3, 1062.4)	755.8 (695.7, 821.2)	795.0 (697.9, 905.7)	--	90.1 (87.0, 92.7)	89.5 (86.0, 92.3)	87.0 (81.4, 91.4)	93.6 (91.0, 95.7)
**Lot** **Consistency**	36.1 (36.1, 36.1)	--	1307.3 (1214.8, 1406.7)	1113.4 (1029.5, 1204.1)	1101.1 (986.3, 1229.3)	--	95.0 (93.2, 96.4)	94.4 (92.5, 96.0)	93.6 (90.5, 96.0)	98.1 (96.9, 98.9)
**Total**	93.7 (90.5, 97.1)	--	1045.6 (1005.6, 1087.1)	752.0 (712.6, 793.6)	697.4 (653.0, 744.8)	--	93.9 (92.9, 94.7)	90.1 (88.6, 91.5)	86.1 (83.8, 88.2)	96.1 (95.2, 96.8)
**PRNT GMTs (95% CI)**						**PRNT Seroresponse four-fold Increase from Baseline**				
**FLW**	35.2 (34.9, 35.5)	102.9 (97.8, 108.3)	162.2 (153.9, 170.9)	121.4 (101.3, 145.6)	--	33.2 (30.3, 36.3)	58.5 (55.2, 61.7)	42.7 (31.3, 54.6)	--	62.1 (59.1, 65.0)
**PREVAIL**	36.5 (35.0, 38.1)	--	123.1 (112.5, 134.7)	85.3 (78.5, 92.7)	107.8 (99.1, 117.3)	--	41.4 (36.6, 46.2)	29.4 (25.2, 34.0)	38.6 (34.0, 43.4)	54.7 (49.8, 59.5)
**STRIVE**	35.6 (35.0, 36.3)	--	122.8 (112.89, 133.6)	103.7 (95.2, 113.0)	147.3 (127.0, 170.8)	--	41.6 (36.6, 46.7)	36.8 (31.6, 42.2)	51.5 (43.6, 59.4)	55.2 (50.2, 60.1)
**Lot** **Consistency**	35.5 (35.5, 36.0)	--	211.7 (198.1, 226.3)	271.8 (253.4, 291.5)	278.3 (251.8, 307.5)	--	64.9 (61.4, 68.3)	74.8 (71.5, 78.0)	76.3 (71.5, 80.7)	83.3 (80.5, 85.9)
**Total**	35.6 (35.3, 35.9)	--	159.3 (153.8, 165.1)	149.7 (142.4, 157.3)	158.1 (148.4, 168.5)	--	54.9 (53.0, 56.9)	52.6 (50.1, 55.1)	54.8 (51.6, 58.0)	65.4 (54.7, 67.2)

CI: confidence interval; EU: ELISA units; FLW: Guinea Front Line Worker trial, PREVAIL: Partnership for Research on Ebola Virus in Liberia; GMTs: geometric mean titers; GP-ELISA: glycoprotein-enzyme-linked immunosorbent assay; PRNT: plaque reduction neutralization test; STRIVE: Sierra Leone Trial to Introduce a Vaccine against Ebola.

## Data Availability

Not applicable.
